# Dislocation angle predicts complications following mandibular condylar fracture treatment: a retrospective cohort study

**DOI:** 10.3389/froh.2026.1812421

**Published:** 2026-04-24

**Authors:** Ingrid Winkler, Chiara Costantini, Babak Saravi, Lara Schorn, Julian Lommen, Felix Schrader, Max Wilkat, Norbert Kübler, Christoph Sproll, Daman Deep Singh

**Affiliations:** 1 Department of Oral and Maxillofacial Plastic Surgery, University Hospital Cologne, Cologne, Germany; 2 Department of Oral, Maxillofacial and Facial Plastic Surgery, Medical Faculty and University Hospital Düsseldorf, Heinrich-Heine-University Düsseldorf, Düsseldorf, Germany

**Keywords:** complications, dislocation angle, hardware removal, mandibular condyle fracture, maxillofacial trauma, open reduction internal fixation, retrospective cohort study, risk factors

## Abstract

**Background:**

Mandibular condylar fractures are among the most common maxillofacial injuries, yet predictors of postoperative complications remain poorly defined. The role of radiographic dislocation angle as a prognostic factor has received limited attention. This study aimed to identify risk factors for complications following condylar fracture treatment, with particular focus on the predictive value of the dislocation angle.

**Methods:**

This retrospective cohort study included 184 patients with mandibular condylar fractures treated at a tertiary referral center between 2009 and 2022. Complications were analyzed according to treatment approach (surgical vs. conservative), and potential risk factors were evaluated using univariate and multivariate logistic regression. The dislocation angle was measured on preoperative imaging and stratified into categories (<60°, 60–89°, ≥90°).

**Results:**

The overall complication rate was 29.0% (49/169), predominantly comprising minor events (17.8%), with major complications occurring in only 7.7%. In the surgical subgroup with available angle measurements (*n* = 91), dislocation angle was significantly associated with complications both in univariate (*p* = 0.049) and multivariate analysis (OR 1.27 per 10°, 95% CI 1.04–1.57, *p* = 0.021). Patients with angles <60° had a complication rate of 5.9% compared to 36.0–42.9% for angles ≥60° (*p* = 0.007). Hardware removal was performed in 43.1% of surgically treated patients.

**Conclusions:**

Preoperative dislocation angle is an independent predictor of complications following condylar fracture treatment. A preliminary threshold of approximately 60° may serve as a candidate parameter for risk stratification and patient counseling, pending prospective validation.

## 1 Introduction

Fractures of the mandibular condyle represent one of the most common injuries in maxillofacial trauma, accounting for 25%–40% of all mandibular fractures (1, 2). The unique anatomy of the temporomandibular joint and the indirect mechanism of injury through force transmission from the chin region make the condylar process particularly susceptible to fracture (3). Due to the critical role of the condyle in mandibular function, mastication, and facial symmetry, appropriate management of these injuries is essential to prevent long-term morbidity (4).

The epidemiology of condylar fractures has remained relatively stable over recent decades. Falls and assaults constitute the predominant etiologies, with a marked male predominance reported across multiple studies (5, 6). Young adults are most commonly affected, though the injury pattern varies with age and mechanism of trauma (7). Bilateral condylar fractures and associated parasymphyseal fractures frequently occur due to the contrecoup mechanism of injury transmission (8).

Despite the high incidence of condylar fractures, their optimal management remains a subject of ongoing debate, with evidence supporting both open reduction and internal fixation (ORIF) and closed reduction with maxillomandibular fixation (9–14). Regardless of the treatment approach chosen, complications including sensory disturbances, facial nerve dysfunction, malocclusion, and pseudarthrosis can significantly impact patients’ quality of life and may require secondary interventions (15–17). Identifying preoperative predictors of such complications is crucial for risk stratification and informed patient counseling. While previous studies have investigated risk factors including fracture displacement, bilateral involvement, and associated mandibular fractures, results have been inconsistent (18, 19). Notably, the degree of condylar fragment dislocation—a readily measurable radiographic parameter—has received surprisingly little attention as a quantitative predictor. From a biomechanical standpoint, greater displacement of the condylar fragment implies increased disruption of the pterygoid musculature, a more complex surgical challenge requiring extensive dissection, and potentially more forceful reduction maneuvers, all of which may predispose to adverse outcomes (3, 13, 14). Yet, no established angular threshold exists that delineates low-risk from high-risk fractures.

The primary aim of this study was to evaluate the predictive value of the preoperative dislocation angle for complications following mandibular condylar fracture treatment. Secondary aims included identifying additional risk factors for adverse outcomes, characterizing complication patterns, and assessing the frequency and indications of hardware removal.

## 2 Methods

### 2.1 Study design and setting

This retrospective cohort study was conducted at the Department of Oral and Maxillofacial Plastic Surgery, University Hospital Düsseldorf, Germany. The study protocol was approved by the local ethics committee (Ethics Committee of the Medical Faculty, Heinrich Heine University Düsseldorf; approval number: 2023-2669). Due to the retrospective design and the use of anonymized routine clinical data, the local ethics committee waived the requirement for individual informed consent. All procedures were performed in accordance with the ethical standards of the institutional research committee and with the 1964 Helsinki Declaration and its later amendments.

### 2.2 Patient selection

#### 2.2.1 Inclusion criteria

- Patients with radiologically confirmed condylar fractures of the mandible
- Treatment between May 2009 and December 2022
- Complete documentation of fracture laterality

#### 2.2.2 Exclusion criteria

- Pathological fractures (tumor-associated)
- Fractures of the condylar head (capitulum) without involvement of the condylar neck
- Incomplete medical records
- Isolated mandibular fractures without condylar involvement

### 2.3 Data collection

Patient data were extracted from electronic medical records and included:

#### 2.3.1 Demographic variables

- Age at time of injury (years)
- Sex (male/female)
- Etiology of fracture (fall/trauma, assault, traffic accident, other)

#### 2.3.2 Fracture characteristics

- Laterality (right, left, bilateral)
- Fracture multiplicity (isolated condylar fracture vs. multiple mandibular fractures)
- Associated mandibular fractures (parasymphyseal, symphyseal, angle, body, ramus)
- Spiessl and Schroll classification (Type I–VI)
- Dislocation angle measured on preoperative computed tomography using 3D multiplanar reconstructions (degrees)

#### 2.3.3 Treatment variables

- Treatment approach (surgical, conservative, combined)
- Surgical approach (retromandibular transparotideal, preauricular, intraoral, other)
- Osteosynthesis material (Medartis, KLS-Martin, Synthes)
- Number of plates used for fixation
- Plate type (miniplate, delta plate, other)

#### 2.3.4 Outcome variables

- Occurrence of complications (yes/no)
- Type of complication
- Hardware removal (yes/no) and time to removal
- Revision surgery (yes/no)

### 2.4 Definitions

A condylar fracture was defined as a fracture involving the condylar neck (collum mandibulae), diagnosed by computed tomography; fractures isolated to the condylar head (capitulum) were excluded. Complications were defined as any adverse event occurring during the postoperative period requiring intervention or documentation, including sensory disturbances, facial nerve dysfunction, wound-related complications (infection, abscess, salivary fistula, dehiscence), pseudarthrosis, malocclusion, and hardware failure. For severity stratification, complications were classified as minor (transient or self-limiting) or major (requiring revision or causing persistent impairment). Condylar fractures were classified according to the Spiessl and Schroll system, comprising Types I–VI based on dislocation severity and fragment contact (20).

The dislocation angle, also referred to as the angle of displacement, is the angle between the long axis of the displaced condylar fragment and the long axis of the mandibular ramus, measured on preoperative CT using 3D multiplanar reconstructions identifying the plane of maximum displacement. A higher angle indicates greater displacement.

### 2.5 Treatment protocol

#### 2.5.1 Surgical treatment

Indications for surgical treatment included:

- Dislocation with loss of ramus height >2 mm
- Dislocation angle >10°
- Bilateral condylar fractures with loss of posterior facial height
- Condylar fractures with concomitant mandibular fractures requiring open reduction
- Failed conservative treatment

Open reduction and internal fixation (ORIF) was performed using the retromandibular transparotideal, preauricular, or intraoral approach based on fracture location and surgeon preference. Fixation was achieved using titanium miniplates (1.5–2.0 mm) with monocortical screws.

#### 2.5.2 Conservative treatment

Indications for conservative treatment included:

- Minimally displaced fractures (dislocation angle <10°)
- Condylar head fractures in adults
- Patient refusal of surgery
- Medical contraindications to general anesthesia

Conservative treatment consisted of maxillomandibular fixation (MMF) using arch bars or intermaxillary fixation screws for 1–3 weeks, followed by functional therapy with guiding elastics and physiotherapy.

#### 2.5.3 Antibiotic prophylaxis

Perioperative antibiotic prophylaxis (single-shot) with amoxicillin/clavulanic acid (2.2 g intravenously) or clindamycin (600 mg intravenously) in case of penicillin allergy was administered at the discretion of the treating surgeon. Postoperative oral antibiotics were prescribed based on individual clinical assessment.

### 2.6 Statistical analysis

Statistical analyses were performed using Python 3.11 with SciPy and scikit-learn libraries. Descriptive statistics were calculated as mean ± standard deviation (SD) for normally distributed continuous variables, median with interquartile range (IQR) for non-normally distributed variables, and frequencies with percentages for categorical variables. Normal distribution was assessed using the Shapiro–Wilk test.

Chi-square test or Fisher's exact test was used for categorical variables. Student's *t*-test was used for normally distributed continuous variables. Mann–Whitney *U* test was used for non-normally distributed continuous variables. For patients with bilateral fractures, the maximum dislocation angle across both sides was used for analysis. The Cochran–Armitage trend test was used to assess the dose–response relationship across ordered dislocation angle categories. In the severity stratification analysis, patients with both minor and major complications were classified under the highest severity category. Binary logistic regression was performed to identify independent risk factors for complications. Variables with *p* < 0.20 in univariate analysis or with established clinical relevance were included in the multivariate model. Results are presented as odds ratios (OR) with 95% confidence intervals (CI). Subgroup analyses were performed for:

- Surgically treated patients (dislocation angle analysis)
- Hardware removal and revision surgery rates

No adjustment for multiple testing was performed due to the exploratory nature of subgroup analyses. No formal sample size calculation was performed due to the retrospective nature of the study. All eligible patients within the study period were included. A two-sided *p*-value < 0.05 was considered statistically significant.

## 3 Results

### 3.1 Study population

Between May 2009 and December 2022, a total of 1,039 patients with mandibular fractures were screened at the Department of Oral and Maxillofacial Plastic Surgery, University Hospital Düsseldorf, Germany. After applying inclusion and exclusion criteria, 184 patients with condylar fractures were included in the final analysis.

### 3.2 Patient demographics and fracture characteristics

The study population comprised 133 males (72.3%) and 51 females (27.7%) with a mean age of 37.5 ± 18.7 years (range: 4–87 years). The most common etiology was fall or trauma (94 patients, 51.1%), followed by assault (55 patients, 29.9%). Traffic accidents accounted for only 1.1% of cases (Table 1).

**Table 1:** Patient demographics and fracture characteristics (*n* = 184).

Variable	*n* (%)	Mean ± SD
Age (years)		37.5 ± 18.7
Range		4–87
Sex		
Male	133 (72.3%)	
Female	51 (27.7%)	
Etiology		
Fall/Trauma	94 (51.1%)	
Assault	55 (29.9%)	
Traffic accident	2 (1.1%)	
Unknown	33 (17.9%)	
Fracture laterality		
Right	74 (40.2%)	
Left	74 (40.2%)	
Bilateral	36 (19.6%)	
Fracture type		
Isolated	63 (34.2%)	
Multiple	121 (65.8%)	
Associated fractures		
Parasymphyseal	74 (40.2%)	
Symphyseal	15 (8.2%)	
Angle	11 (6.0%)	
Condylar head	9 (4.9%)	

Regarding fracture laterality, unilateral fractures were evenly distributed between the right (74 patients, 40.2%) and left side (74 patients, 40.2%), while bilateral condylar fractures were present in 36 patients (19.6%). Multiple mandibular fractures were observed in 121 patients (65.8%), with parasymphyseal fractures being the most common associated injury (74 patients, 40.2%).

Spiessl and Schroll classification data were available for 73 fractures on the right side and 78 fractures on the left side. Type V fractures were most prevalent (right: 34.2%, left: 39.7%), followed by Type I (right: 35.6%, left: 29.5%) and Type IV fractures (right: 24.7%, left: 25.6%). The mean dislocation angle measured on preoperative imaging was 85.4° ± 29.5° for right-sided and 82.2° ± 27.5° for left-sided fractures.

### 3.3 Treatment

Treatment data were available for 172 patients. Surgical management was performed in 139 patients (80.8%), while 29 patients (16.9%) were treated conservatively, and 4 patients (2.3%) received combined treatment (Table 2).

**Table 2:** Treatment details.

Variable	*n* (%)
Treatment approach (*n* = 172)	
Surgical	139 (80.8%)
Conservative	29 (16.9%)
Combined	4 (2.3%)
Surgical approach (retromandibular)	
Right side	52/68 (76.5%)
Left side	49/63 (77.8%)
Osteosynthesis material (*n* = 100)	
Medartis^®^	82 (82.0%)
KLS-Martin^®^	10 (10.0%)
Synthes^®^	6 (6.0%)
Number of plates per side	
1 plate	25/117 (21.4%)
2 plates	89/117 (76.1%)
3 plates	3/117 (2.6%)

Among surgically treated patients, the retromandibular transparotideal approach was the predominant surgical access, used in 76.5% of right-sided and 77.8% of left-sided fractures. Medartis osteosynthesis material was used in 82.0% of cases, followed by KLS-Martin (10.0%) and Synthes (6.0%). Two-plate fixation was the most common technique, applied in 76.1% of cases.

### 3.4 Antibiotic therapy

Antibiotic therapy data were available for 169 patients. Perioperative antibiotics were administered to 37 patients (21.9%), while postoperative antibiotics were given to 81 patients (47.9%). The distribution of antibiotic regimens was as follows: no antibiotics (75 patients, 44.4%), postoperative only (57 patients, 33.7%), peri- and postoperative (24 patients, 14.2%), and perioperative only (13 patients, 7.7%).

### 3.5 Complications

#### 3.5.1 Overall complication rate

Complication data were available for 169 patients. Overall, 49 patients (29.0%) experienced at least one complication (Table 3). The complication rate was 30.1% (40/133) in the surgical group and 24.1% (7/29) in the conservative group. This difference was not statistically significant (Fisher's exact test, *p* = 0.653). Seven patients who received combined treatment (*n* = 4) or had unavailable treatment data (*n* = 3) are included in the overall rate but not in either subgroup.

**Table 3:** Complications by treatment approach.

Variable	Total (*n* = 169)	Surgical (*n* = 133)	Conserv. (*n* = 29)	*p*-value
Overall complication rate	49 (29.0%)	40 (30.1%)	7 (24.1%)	0.653†
Complication types*				
Sensory disturbance	14 (28.6%)	12	2	
Facial nerve dysfunction	9 (18.4%)	8	0	
Wound-related	13 (26.5%)	12	0	
Pseudarthrosis	5 (10.2%)	1	4	
Malocclusion	4 (8.2%)	3	1	

* Percentages in the total column are calculated from patients with complications (*n* = 49). Subgroup counts are absolute numbers; they do not sum to the total column because 7 patients with combined treatment (*n* = 4) or unavailable treatment data (*n* = 3) are included in the total but not shown in either subgroup column.

† Fisher's exact test.

#### 3.5.2 Complication types

**Figure 1:**
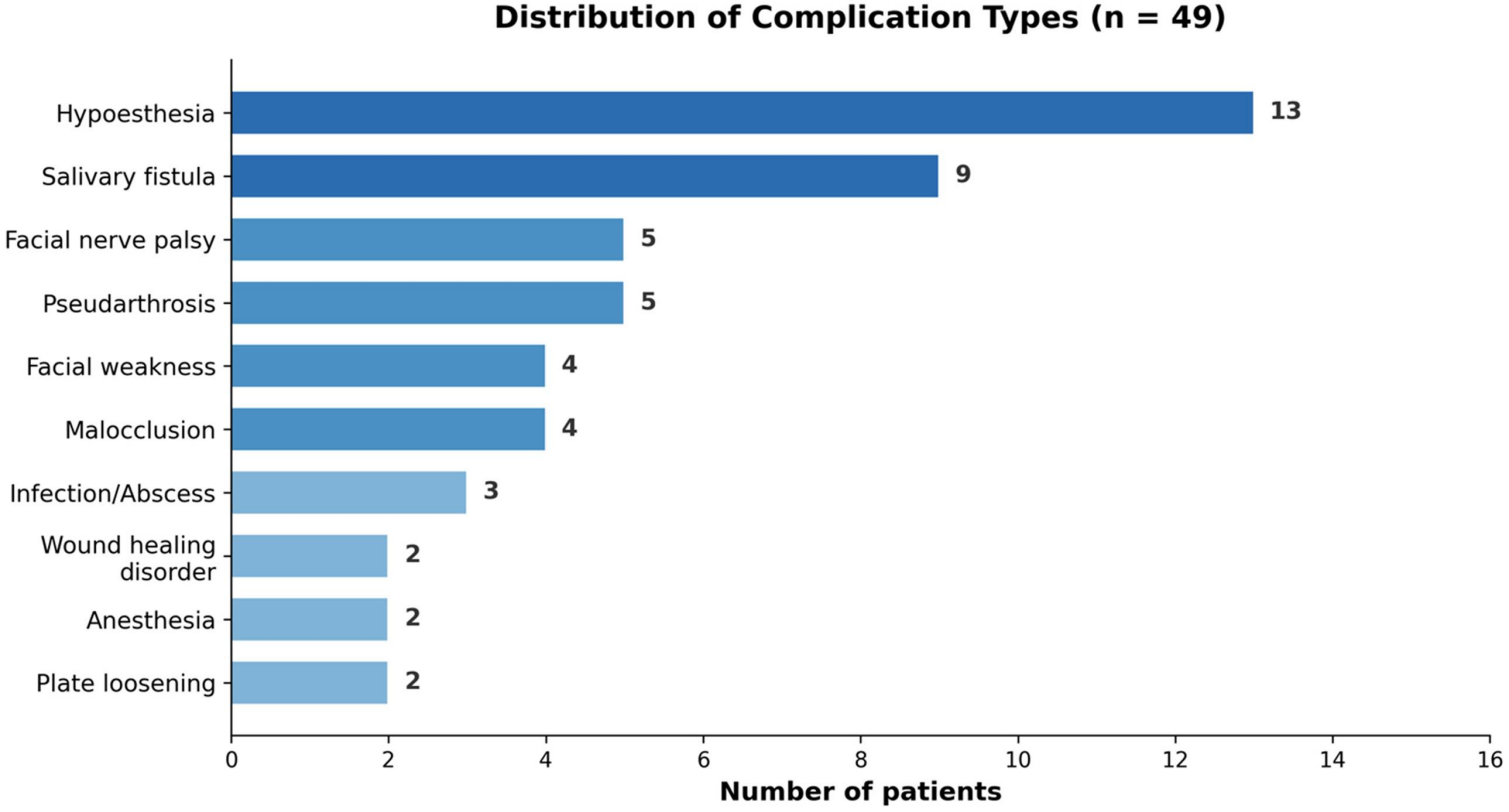
Distribution of complication types among 49 patients with complications. Sensory disturbances (hypoesthesia) were the most common adverse event, followed by salivary fistula and facial nerve dysfunction.

Among the 49 patients with complications, sensory disturbances were the most frequent adverse event, occurring in 14 patients (28.6%), with hypoesthesia being the predominant subtype (13 patients, 26.5%). Wound-related complications were observed in 13 patients (26.5%), including salivary fistula (9 patients, 18.4%) and infection or abscess (3 patients, 6.1%). Facial nerve dysfunction occurred in 9 patients (18.4%), comprising facial nerve palsy (5 patients, 10.2%) and transient facial weakness (4 patients, 8.2%). Pseudarthrosis was documented in 5 patients (10.2%), and malocclusion in 4 patients (8.2%) (Figure 1).

When stratified by clinical severity, minor complications (predominantly sensory disturbances and transient facial nerve dysfunction) occurred in 30 patients (17.8%), while major complications requiring intervention (pseudarthrosis, malocclusion, infection, hardware failure) were observed in 13 patients (7.7%) (Table 4). This distinction is clinically relevant, as the overall complication rate of 29% is predominantly driven by minor, often self-limiting events.

**Table 4:** Stratification of complications by severity (*n* = 169).

Complication Category	*n*	Rate (%)
No complication	120	71.0%
Minor complications	30	17.8%
Hypoesthesia	11	6.5%
Salivary fistula	9	5.3%
Facial nerve palsy (transient)	5	3.0%
Facial weakness (transient)	4	2.4%
Anesthesia	2	1.2%
Wound healing disturbance	1	0.6%
Major complications	13	7.7%
Pseudarthrosis	5	3.0%
Malocclusion	4	2.4%
Infection/Abscess	3	1.8%
Plate loosening	2	1.2%
Other/Unknown	6	3.6%

Minor: transient or self-limiting events. Major: events requiring revision or causing persistent impairment. Patients with both minor and major complication types were classified under major; consequently, subtype counts within the minor category may be lower than the total patient counts reported in Figure 1 (e.g., 2 of 13 hypoesthesia patients also had major complications and are counted under major).

### 3.6 Risk factor analysis

#### 3.6.1 Univariate analysis

Univariate analysis was performed to identify potential risk factors for complications (Table 5). Patient age (39.1 ± 19.1 vs. 37.4 ± 18.5 years, *p* = 0.534), male sex (31.5% vs. 22.2%, *p* = 0.329), bilateral fracture involvement (33.3% vs. 27.9%, *p* = 0.690), multiple fractures (29.8% vs. 23.2%, *p* = 0.324), and surgical treatment (30.1% vs. 24.1%, *p* = 0.653) were not significantly associated with complication occurrence.

**Table 5:** Univariate analysis of risk factors for complications.

Variable	Complication (*n* = 49)	No complication (*n* = 120)	*p*-value
Age (years), mean ± SD	39.1 ± 19.1	37.4 ± 18.5	0.534
Male sex, *n* (%)	39 (31.5%)	85 (68.5%)	0.329
Bilateral fracture, *n* (%)	11 (33.3%)	22 (66.7%)	0.690
Multiple fractures, *n* (%)	36 (31.9%)	77 (68.1%)	0.324
Surgical treatment, *n* (%)	40 (30.1%)	93 (69.9%)	0.653
Postoperative antibiotics, *n* (%)	27 (34.2%)	52 (65.8%)	0.215
Surgical subgroup (*n* = 91)			
Dislocation angle (°), mean ± SD	93.4 ± 17.4	79.1 ± 31.1	**0** **.** **049** *
<60°	1/17 (5.9%)	16/17 (94.1%)	***p* for trend = 0.007 (Cochran-Armitage)**
60–89°	9/25 (36.0%)	16/25 (64.0%)	
≥90°	21/49 (42.9%)	28/49 (57.1%)	

* Statistically significant (*p* < 0.05).

Neither perioperative (34.3% vs. 27.6%, *p* = 0.571) nor postoperative antibiotic therapy (34.2% vs. 24.1%, *p* = 0.215) showed a significant protective effect against complications.

#### 3.6.2 Dislocation angle as a predictor (key finding)

**Figure 2:**
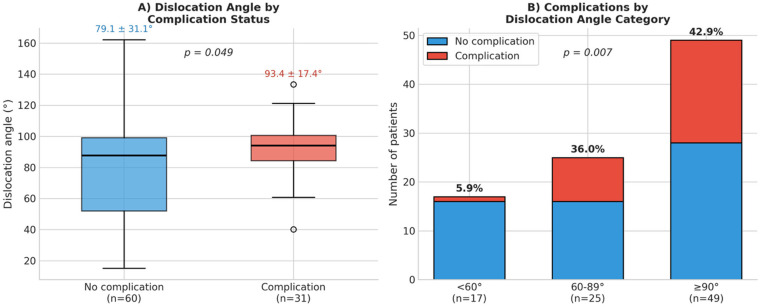
Association between dislocation angle and complications in surgically treated patients. **(A)** Box plot comparing dislocation angles in patients with and without complications (*p* = 0.049). **(B)** Complication rates stratified by dislocation angle categories showing a significant dose-response relationship [*p* for trend = 0.007 (Cochran-Armitage)].

In the surgical subgroup with available dislocation angle measurements (*n* = 91), a significant association was found between the degree of dislocation and complication occurrence. Patients who developed complications had a significantly higher mean dislocation angle compared to those without complications (93.4° ± 17.4° vs. 79.1 ± 31.1°, *p* = 0.049) (Figure 2A). When stratified by dislocation angle categories, a clear dose-response relationship emerged: patients with dislocation angles <60° had a complication rate of only 5.9% (1/17), while those with angles between 60 and 89° had a rate of 36.0% (9/25), and patients with angles ≥90° had a rate of 42.9% (21/49) (*p* = 0.007) (Figure 2B).

### 3.7 Multivariate analysis

**Figure 3:**
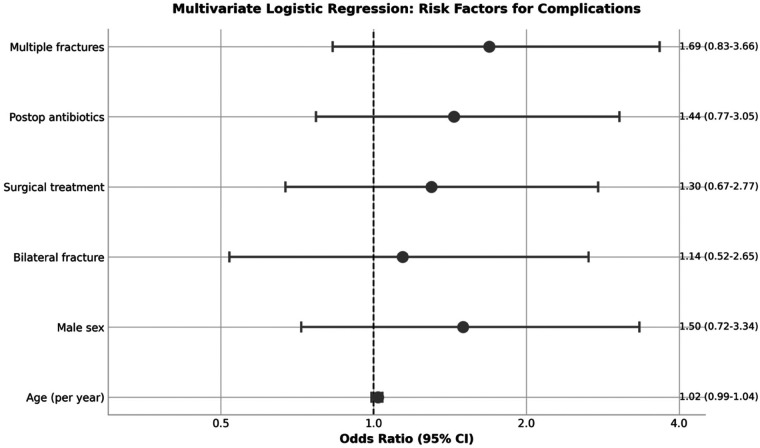
Forest plot showing odds ratios from multivariate logistic regression analysis. None of the analyzed variables reached statistical significance as independent predictors of complications.

Multivariate logistic regression analysis including age, sex, bilateral involvement, surgical treatment, postoperative antibiotic use, and multiple fractures was performed in 161 patients with complete data (Table 6). None of the analyzed variables reached statistical significance as independent predictors of complications. Multiple fractures showed the highest odds ratio (OR: 1.69, 95% CI: 0.83–3.66, *p* = 0.156), followed by male sex (OR: 1.50, 95% CI: 0.72–3.34, *p* = 0.298) and postoperative antibiotic use (OR: 1.44, 95% CI: 0.77–3.05, *p* = 0.267) (Figure 3).

**Table 6:** Multivariate logistic regression analysis for complications.

Variable	Odds Ratio	95% CI	*p*-value
Age (per year)	1.02	0.99–1.04	0.182
Male sex	1.50	0.72–3.34	0.298
Bilateral fracture	1.14	0.52–2.65	0.742
Surgical treatment	1.30	0.67–2.77	0.453
Postoperative antibiotics	1.44	0.77–3.05	0.267
Multiple fractures	1.69	0.83–3.66	0.156

Model based on 161 patients with complete data.

To further evaluate the independent predictive value of dislocation angle, a separate multivariate analysis was performed within the surgical subgroup with available angle measurements (*n* = 91, 31 events). After adjusting for age, sex, bilateral fracture involvement, multiple fractures, and postoperative antibiotic use, dislocation angle remained significantly associated with complications (OR: 1.27 per 10° increase, 95% CI: 1.04–1.57, *p* = 0.021) (Table 7). This confirms that the association between dislocation angle and complications is independent of other clinical variables.

**Table 7:** Multivariate logistic regression analysis in surgical subgroup (*n* = 91).

Variable	OR	95% CI	*p*-value
Age (per year)	1.00	0.97–1.03	0.924
Male sex	1.67	0.47–5.87	0.426
Bilateral fracture	0.61	0.17–2.12	0.433
Multiple fractures	1.61	0.55–4.75	0.389
**Dislocation angle (per 10°)**	**1** **.** **27**	**1.04** **–** **1.57**	**0** **.** **021** *
Postoperative antibiotics	1.38	0.53–3.65	0.510

* *p* < 0.05; CI, confidence interval; OR, odds ratio.

Model statistics: Pseudo R² = 0.075, AIC = 122.0.

Bold values indicate statistical significance (*p* < 0.05).

### 3.8 Hardware removal

Hardware removal data were available for 167 patients. Hardware removal was performed in 74 patients (44.3%) at a mean interval of 325 ± 204 days (median: 274 days, IQR: 204–366 days) postoperatively, corresponding to approximately 10.7 months (Table 8). Among surgically treated patients specifically, the removal rate was 43.1% (59/137).

**Table 8:** Hardware removal and revision surgery.

Variable	*n* (%)	Details
Hardware removal (*n* = 167)		
Performed	74 (44.3%)	
Not performed	93 (55.7%)	
Time to removal (*n* = 68)		
Mean ± SD		325 ± 204 days
Median (IQR)		274 (204–366) days
Indication for removal*		
Consolidation	10 (40.0%)	
Patient request	7 (28.0%)	
Pseudarthrosis	2 (8.0%)	
Other	6 (24.0%)	
Revision surgery (*n* = 168)	11 (6.5%)	

* Calculated from documented cases only (*n* = 25).

**Figure 4:**
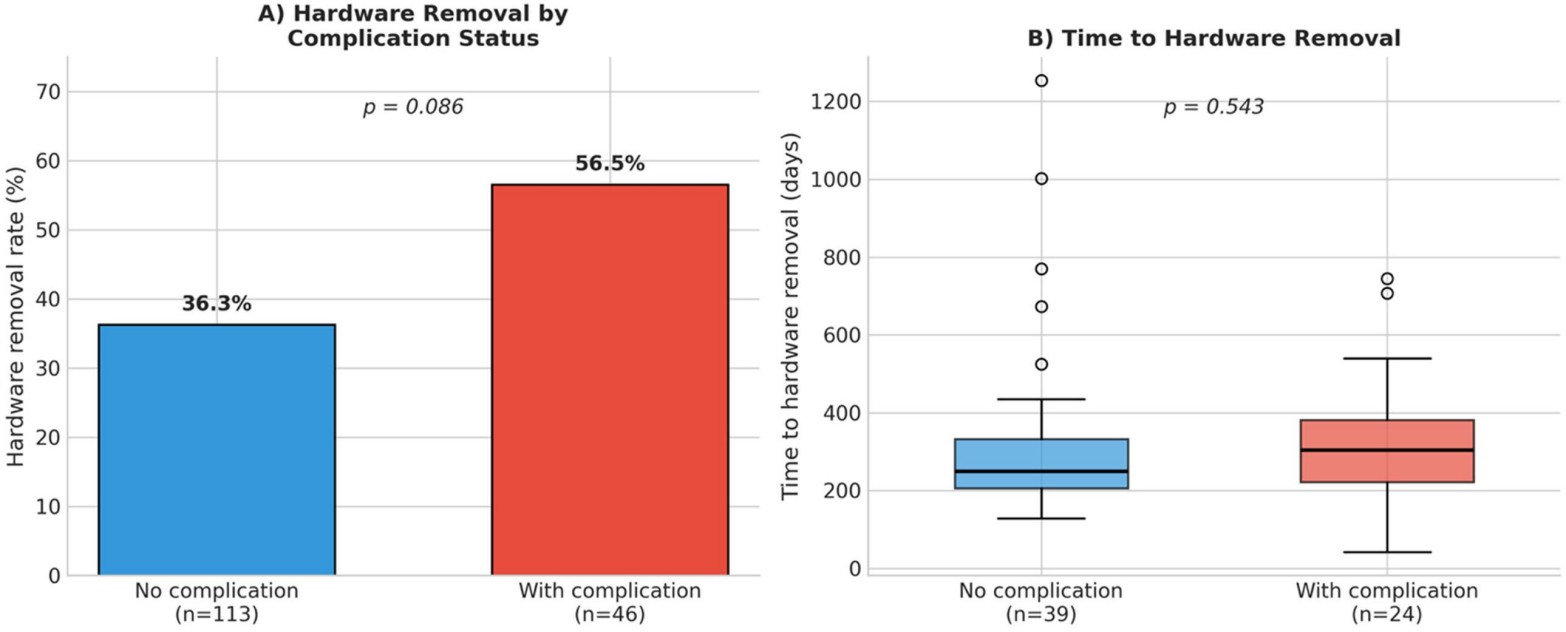
Hardware removal analysis. **(A)** Hardware removal rate by complication status showing a trend toward higher removal rates in patients with complications (56.5% vs. 36.3%, *p* = 0.086). **(B)** Time to hardware removal stratified by complication status showing no significant difference between groups (median 304 vs. 250 days, *p* = 0.543).

To assess whether complications influenced the decision for hardware removal, we analyzed the relationship between complication occurrence and subsequent plate removal (Figure 4). Patients who experienced complications had a higher rate of hardware removal compared to those without complications (56.5% vs. 36.3%), although this difference did not reach statistical significance (OR: 2.09, 95% CI: 0.91–4.80, *p* = 0.086).

The time interval to hardware removal did not differ significantly between patients with complications (328 ± 168 days, median: 304 days) and those without (327 ± 231 days, median: 250 days; *p* = 0.543). Revision surgery was required in 11 of 168 patients (6.5%) with available follow-up data. Among patients who underwent revision surgery, 6 (54.5%) subsequently required hardware removal at a later time point (mean interval: 327 days after revision). The remaining 5 patients with revision surgery did not undergo hardware removal during the follow-up period.

## 4 Discussion

This retrospective study of 184 patients with mandibular condylar fractures identified the preoperative dislocation angle as a significant predictor of complications. Patients with dislocation angles ≥60° demonstrated substantially higher complication rates compared to those with angles <60° (36.0–42.9% vs. 5.9%, *p* = 0.007), and this association remained significant after multivariate adjustment (OR: 1.27 per 10°, 95% CI: 1.04–1.57, *p* = 0.021). The overall complication rate of 29% was predominantly driven by minor, self-limiting events, with major complications requiring intervention occurring in only 7.7% of patients.

The finding that greater condylar fragment displacement predicts adverse outcomes aligns with biomechanical principles and previous reports. Ellis and Throckmorton (13) demonstrated that increased displacement correlates with greater disruption of the pterygoid musculature attachment, potentially compromising fragment stability. A larger dislocation angle, especially exceeding our identified threshold of 60°, likely reflects a more complex surgical challenge. Severe displacement often requires more extensive soft tissue dissection and more forceful manipulation of the proximal fragment to achieve anatomical reduction. This increased surgical trauma may explain the higher incidence of complications, such as transient facial nerve palsy or surgical site infections, observed in our high-angle cohort (13). Accordingly, Sawazaki et al. (3) reported that severely displaced fractures were associated with prolonged recovery and increased temporomandibular dysfunction. Our data extend these observations by suggesting a candidate quantitative threshold: a dislocation angle of approximately 60° may represent a clinically meaningful cut-off. However, given the retrospective design, the partial availability of angle measurements (67.6% of surgical patients), and the absence of formal inter- and intra-observer reliability testing, this value should be regarded as preliminary and hypothesis-generating rather than as a definitive cut-off, and requires confirmation in prospective studies before being adopted in clinical practice. The overall complication rate is comparable to published rates of 15%–35% (2, 10). However, stratification by severity provides important context. The major complication rate of 7.7%—comprising pseudarthrosis, malocclusion, infection, and hardware failure—is consistent with contemporary series employing modern fixation techniques (9). The predominance of minor complications, particularly sensory disturbances and transient facial nerve dysfunction, reflects anatomical challenges inherent to condylar surgery rather than treatment failure. Most neurological complications were transient, consistent with recovery patterns reported by others (21, 22).

We observed no significant difference in complication rates between surgically (30.1%) and conservatively (24.1%) treated patients (Fisher's exact test, *p* = 0.653). These rates are consistent with previous reports showing comparable complication frequencies between treatment modalities, with overall rates ranging from 15% to 35% across both surgical and conservative approaches (23, 24). However, this comparison is limited by confounding by indication, as surgically treated patients typically presented with more severe injuries. This limitation is common to retrospective studies comparing treatment modalities and precludes conclusions regarding superiority of either approach (25, 26). Hardware removal was performed in 44.3% of patients (43.1% among surgically treated patients specifically), predominantly for routine consolidation or patient request. The decision for hardware removal in maxillofacial trauma remains institution-dependent, with emerging evidence questioning its routine necessity (27–30). Revision surgery was required in 6.5% of patients, which falls within the range reported in contemporary series (17, 31). Neither perioperative nor postoperative antibiotic therapy demonstrated significant protective effects, though prospective trials are needed before modifying current practices.

Several limitations warrant consideration. The retrospective design introduces inherent biases in patient selection and data completeness. Dislocation angle measurements were performed by a single reviewer without formal reliability assessment. Future studies should incorporate inter- and intra-observer reliability testing to validate the reproducibility of dislocation angle measurements across different clinical settings and observers, which would further strengthen the clinical applicability of the proposed 60° threshold. Spiessl and Schroll classification data were available for only 40% of fractures, and dislocation angles for 67.6% of surgical patients, potentially introducing selection bias. The composite outcome included both transient and major complications with potentially different risk profiles. Follow-up duration was heterogeneous, and pediatric patients were not analyzed separately despite known differences in fracture healing capacity (32, 33).

In summary, our study demonstrates three key findings: (1) the preoperative dislocation angle is an independent predictor of complications following condylar fracture treatment, with a tentative threshold of approximately 60° that may help delineate lower- from higher-risk fractures pending prospective validation; (2) the overall complication rate of 29% is predominantly driven by minor, transient events, with major complications occurring in only 7.7% of patients; and (3) hardware removal was performed in 43.1% of surgically treated patients, primarily for routine consolidation or patient request rather than complication-driven necessity. From a clinical perspective, the dislocation angle should not be used to contraindicate surgical treatment, as severely displaced fractures typically require open reduction. Rather, the angle serves as a tool for preoperative risk stratification and informed consent. Patients with dislocation angles ≥60° should be counseled about the higher likelihood of predominantly minor complications and may benefit from closer postoperative monitoring.

## 5 Conclusion

The preoperative dislocation angle is an independent predictor of complications following mandibular condylar fracture treatment. In our cohort, a threshold of approximately 60° was associated with a substantially increased risk of complications, predominantly minor and transient events. Given the retrospective design, the limited availability of angle measurements, and the absence of inter- and intra-observer reliability assessment, this threshold should be considered preliminary and hypothesis-generating. Prospective multicenter studies with standardized measurement protocols and formal reliability testing are required to validate this cut-off and to determine whether targeted perioperative interventions can mitigate complication risk in patients with high dislocation angles.

## Data Availability

The raw data supporting the conclusions of this article will be made available by the authors, without undue reservation.
